# Design and Development of an Edible Coating for a Ready-to-Eat Fish Product

**DOI:** 10.3390/polym16030346

**Published:** 2024-01-27

**Authors:** Ina Bremenkamp, Maria J. Sousa-Gallagher

**Affiliations:** Process & Chemical Engineering, School of Engineering & Architecture, College of Science, Engineering and Food Science, University College Cork, T12 K8AF Cork, Ireland; 117106445@umail.ucc.ie

**Keywords:** edible coating, chitosan, sodium alginate, food quality, shelf life, RTE fish products

## Abstract

The application of chitosan and alginate coatings for a ready-to-eat (RTE) baked fish product was studied. An experimental design was used to investigate the effect of coating a polysaccharide concentration and glycerol addition on the safety (microbial growth) and quality (water loss and lipid oxidation) of an RTE fish product under optimal and abused storage conditions. The results showed that a chitosan coating with 1% (*w*/*v*) chitosan in 1% (*v*/*v*) acetic acid and 15% (*w*/*w* chitosan) glycerol, or a 1% (*w*/*v*) alginate coating with no glycerol and no crosslinking, showed the best performance in controlling the tested safety and quality parameters. The desirability method was used to identify the shelf lives of chitosan, alginate, and double-coated RTE products. The chitosan-coated samples showed the best performance with a three-fold shelf-life extension compared to the uncoated products stored at 4 °C. Moreover, the tested coatings demonstrated their ability to provide protective functions under abused storage conditions. These results strongly suggest that edible coatings have significant potential in enhancing the shelf life and safety of ready-to-eat (RTE) fish products.

## 1. Introduction

Rethinking packaging for recyclability, circularity, and sustainability is required to overcome current packaging challenges, such as fossil fuel raw material dependency and waste management. The use of an edible coating is one possible strategy designed to improve the sustainability of food packages, which combines the need to use renewable natural biomaterials and reduce packaging waste by being edible or biodegradable [1,2]. A coating is a thin homogeneous layer formed from a natural film, such as carbohydrates, proteins, or lipids, on the product surface [3], which acts as a packaging by preventing or controlling the deteriorative reactions of the product. Potential renewable natural film-forming polysaccharides, sourced from the maritime environment, are, for example, chitosan and alginate. The advantages of using maritime polymeric materials as edible coatings for seafood products are their availability in the production area of seafood products and the generation of a new market for processing waste products [4].

Chitosan is a derivative of chitin, the second most abundant natural polymer in nature, and it is mainly produced from shrimp and crab shells, which is a waste product of the seafood industry with a global waste accumulation of 6 to 8 million tons [5,6]. Chitosan is a suitable material for edible coatings as it is nontoxic, biodegradable, biocompatible, and generally recognised as safe by the US Food and Drug Administration [7]. Chitosan coatings have shown excellent coating behaviour by forming a transparent, invisible, and adhesive layer around food products, and they have been shown to control microbial growth [8,9] and reduce fat oxidation [10]. Variations in chitosan coatings’ composition and processing factors have been reported, e.g., chitosan with different degrees of acetylation from 70 to 95%, chitosan concentrations from 0.5 to 3%, different solvents with concentrations from 1 to 2%, stirring temperatures from 23 to 40 °C, or the addition of glycerol from 0.5 to 75% of *w*/*w* chitosan [8,9,11,12,13,14,15,16]. The potential of chitosan coatings is well studied for different fresh fish products [8,11,14,17], but limited studies for ready-to-eat (RTE) food products, particularly RTE seafood products [9,10,12], could be found.

Alginate can be found in the cell walls and intercellular regions of most brown algae species, which are abundant in many coastlines. Alginates are seen as a valuable and sustainable resource, avoiding competition for land use with food production, and they are low-cost products [18]. Alginate is biodegradable, biocompatible, nontoxic, has excellent film-forming properties, and is widely studied as a carrier coating material for natural antimicrobial or antioxidant agents for fresh fish products [19,20,21,22,23,24,25]. Alginate coatings have been reported to control food product degradation processes, such as lipid oxidation [20,21,22,23,24,25] and microbiological growth [17,18,19]. Studies with alginate coatings applied to RTE seafood products are rare [10], and the effect can depend on the coating composition and development process.

RTE seafood products are in high demand considering current consumer trends of convenience, health consciousness, nutrition, mildly preserved foods, and products with an enhanced shelf life and controlled product quality [26]. Additionally, considering the growth in ageing populations in every country in the world [27], seafood products can provide an important food source with high nutritional value [28]. RTE food is defined as food intended for direct human consumption without the need for cooking or other processing preparation steps to eliminate or reduce microorganisms of concern [29]. The shelf life of chilled RTE products is limited, and suitable handling until consumption is crucial to maintain product quality and provide a safe product. The use of hurdle technology, such as, for example, pre-preservation steps, cooled storage, and packaging, is a common strategy applied to provide a product with an extended shelf life. The application of coatings for RTE seafood products is still limited despite having several advantages, such as enhancing product safety and quality and being an alternative edible packaging option.

The objective of this study was to develop an edible coating to control the degradation processes of an RTE baked fish fillet. Two maritime-based coatings, based on chitosan and alginate, were developed and optimised for material properties, coating composition, and the development process. Additionally, the performance of the selected coatings, as well as a bilayer coating designed to combine the advantages of both coatings, were compared under optimal and abused storage conditions. The shelf lives of baked fish products with and without coatings were estimated using a desirability analysis to compare the overall performance of the studied coatings.

## 2. Materials and Methods

### 2.1. Chemicals

Acetic acid ≥ 99.7% (64-19-7), calcium chloride anhydrous ≥ 97% (10043-52-4), chitosan (medium molecular weight) (9012-76-4), glycerol ≥ 99.5% (56-81-5), peptone buffer, plate count agar, sodium alginate (9005-38-3), 1,1,3,3-tetraethoxypropane ≥ 96% (122-31-6), 2-thiobarbituric acid ≥ 98% (504-17-6), and trichloroacetic acid ≥ 99.0% (76-03-9) were purchased from Sigma-Aldrich (Darmstadt, Germany).

### 2.2. Experimental Design and Set Up

An overview of the performed experimental designs is illustrated in Table 1. A 3^2 full factorial design was used to study the effect of chitosan concentration (1, 2, and 3%) and glycerol concentration (0, 15, and 30% *w*/*w* chitosan) on the coating performance to control the degradation processes of a baked fish sample stored at 4 °C for 14 days. The effect of the composition of an alginate coating was studied with a 2^3 full factorial design. The studied factors were alginate concentration (1 or 2% (*w*/*v*)), glycerol concentration (0 or 1.5% (*w*/*w*) alginate) and crosslinking the alginate coating with CaCl_2_ to form calcium alginate (yes/no). Additionally, the performance of the selected chitosan coating, selected alginate coating, and samples coated with a bilayer coating consisting of a chitosan and alginate layer at optimal storage conditions (4 °C) for 21 days and abused storage conditions (14 °C) for 7 days were investigated. All samples were placed into a storage container (KIS C, XS) with a lid to control airflow and stored in an incubator (Sanyo, Osaka, Japan) at defined temperatures. The functionality of the coatings was assessed by measuring the lipid oxidation, water loss and the microbial total aerobic growth at day 0 and defined storage times (2, 4, and 7 days or 7, 14, and 21 days for samples stored at 14 and 4 °C, respectively). An uncoated product was used as reference. All measurements were at least conducted in triplicates, and values presented in this study are average values.

### 2.3. Sample Preparation

**Figure 1:**
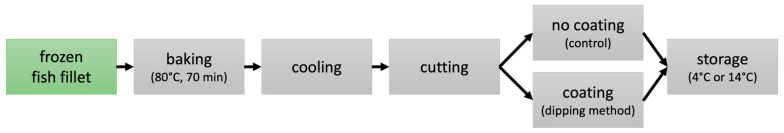
Schematical illustration of processing of fish fillet samples.

Frozen white fish fillet was purchased from local supermarket. The nutritional composition of the product was 0.8% fat, 17% protein, 0.25% salt, and 0.5% fibre. The frozen fillets were placed on a baking tray and prepared with a mild heating treatment in an oven at 80 °C for 70 min (Figure 1). The baked fillets were allowed to cool down to room temperature in a sterile storage box to avoid any cross-contamination before the fillets were cut into pieces of 20 to 25 g. The samples were examined, and pieces with a similar thickness was selected for this study. The average water content of the RTE fish product after baking was 75%. Quality characteristics and microbiological contamination of the samples were analysed at day 0, which was taken as reference point to analyse the deterioration process during storage. The fish samples were randomly selected for each sample category.

### 2.4. Coating Preparation and Application

Chitosan film-forming solutions (FFS) were prepared by either dissolving 1, 2 or 3% (*w*/*v*) chitosan in a 1% (*v*/*v*) acidic solution. The mixture was stirred with a head stirrer (IKA, Staufen, Germany) for 2 h at room temperature. Afterwards, when required, 15 or 30% glycerol (*w*/*w* chitosan powder) was added, and the mixture was stirred for an additional 10 min. Alginate FFSs were prepared by dissolving sodium alginate (1 or 2% *w*/*v*) in distilled water by stirring for 30 min at 80 °C. Afterwards and when required, glycerol was added, and the mixture was stirred for an additional 10 min. The solutions were cooled down to room temperature. Both FFSs were allowed to rest for at least 12 h at 4 °C before use.

Samples were coated by immersing the samples into FFS for 30 s, which was followed by a draining step for 5 min. The process was repeated to ensure a complete coating formation on the sample surface. For the calcium–alginate coating, the sodium alginate-coated samples were immersed into a 0.2 M CaCl_2_ solution. A double coating was applied by first immersing the sample into chitosan FFS, drained for 5 min and afterwards immersed into the alginate FFS. Samples were allowed to air dry during storage.

### 2.5. Characterisation of Coating

Apparent viscosity of the FFS was measured using a Haake Rotor viscometer equipped with a coaxial cylinder geometry at 30 °C ± 0.1. The shear rate was increased from 0.1 to 1000 s^−1^ over 4 min and then held at 1000 s^−1^ for 3 min, after which it was decreased from 100 to 0.1 s^−1^ over 4 min.

The wet coating load (mg liquid coating/cm^2^ sample) defined as the coating adhered to the sample after the coating process was calculated by weighting the samples before and after the coating process. Additionally, the surface area of the samples was manually measured with the aid of a ruler and calculated by assuming a rectangular shape of the fish samples.

### 2.6. Characterisation of Product Quality

Thiobarbituric acid reactive substance assay (TBARS) was used to determine the lipid oxidation of samples [30]. Briefly, a 4 g sample was mixed with 10 mL of 10% trichloroacetic acid (*w*/*v*). Then, 5 mL of control solution, containing an aliquot of 30 μL of a 10^−3^ M 1,1,3,3-tetraethoxypropane (TEP) or 5 mL of distilled water, was added and vortexed for 5 min. Freshly prepared 0.001 M thiobarbituric acid solution was added (5 mL) and mixed for 1 min. The reaction mixture was centrifugated at 3500 rpm for 5 min (Universal 16R, Hettich, Tuttlingen, Germany), and the supernatant was filtered through a Whatman No. 1 filter. Afterwards, the mixture was heated in a water bath (WB14, Memmert, Schwabach, Germany) at 95 °C for 35 min and placed for 10 min into a cooled water bath for cooling. The supernatant absorbance was recorded against a blank, prepared as described above without the addition of sample, at 532 nm using a spectrophotometer (Libra S22, Biochrom, Cambridge, UK). Standard solutions were prepared using 1,1,3,3-tetraethoxypropane to quantify the malondialdehyde (MDA) content, and results were expressed as mg MDA equivalent/kg sample.

The weight of RTE fish samples was measured using a precision balance (Sartorius, Göttingen, Germany). The weight loss (WL) in % was calculated based on the initial (W_0_) and final weight (W_f_) of the product, as follows:

WL = [(W_0_ − W_f_)/W_0_] × 100 (1)

For coated samples, the initial weight was set to the product weight after the coating was applied.

The total aerobic count (TAC) of the product was analysed by aseptically transferring around 10–15 g of sample to a sterile stomacher bag (Sewards Ltd., Worthing, UK) and adding peptone buffer to achieve a 10-fold dilution. The sample was mashed and hand shacked for 2 min. Appropriate dilutions were prepared of the resulting suspension with serially dilution in peptone buffer. For the total microbial count, the pour plate method with a plate count agar (PCA, Sigma Aldrich) was applied. The agar plates were incubated at 30 °C for 72 h [8]. Results were expressed as log10 colony-forming units per gram sample (log CFU/g).

### 2.7. Statistical Analysis

Analysis of Variance (ANOVA) and post hoc analysis using Tukey’s test at a 95% confidence level was used to assess significant differences between the tested coating formulations. Full factorial designs were analysed with a significance level of 5%. Additionally, the desirability was calculated [31] as follows:

D = d_1_^w1^ × d_2_^w2^ × … × d_n_^wn^ (2)

where, D is the total desirability, d is the desirability score of each property studied and w is the weight conferred to each property. Each property was weighted equally. The desirability score is determined using a linear transformation to the values from the Pareto front, scaling the objectives between 0 and 1 from worst to best quality. The optimal combination of the tested coatings was validated qualitatively using characteristic quality and safety properties describing a lower level of product degradation, namely low lipid oxidation, water loss and TAC during storage. In order to avoid the elimination of some results that could be important to explain some observed phenomena, results with 0.05 < *p* < 0.1 were considered marginally significant and also considered to the analysis [32]. The statistical analysis was performed by using Statistica software for Windows v. 7.1 (Tulsa, OK, USA).

## 3. Results

The physical and protective properties of edible coatings can be influenced by factors such as the coating material concentration, coating formulation, and the addition of plasticisers as additives. Therefore, the initial focus was on identifying a suitable coating formulation for a ready-to-eat (RTE) seafood product, using chitosan and alginate. The performance of the coating was evaluated by examining critical quality parameters in RTE baked fish fillets stored for 14 days at 4 °C. Lipid oxidation, a significant factor limiting the shelf life of muscle foods due to the presence of higher amounts of unsaturated fatty acids, was measured to assess off-flavour, colour and odour development, and it also contributes to texture deterioration [7,33]. The malondialdehyde content, indicative of the degree of secondary lipid oxidation developed during storage [34], was used as the first critical quality parameter. Additionally, controlling weight loss during storage was deemed crucial for determining shelf life, as excessive weight loss can alter the product appearance and reduce consumer acceptance. The third critical quality parameter involved determining the total aerobic count to assess the overall safety and quality of the RTE fish product. RTE food products which undergo only mild heat treatment and are distributed at cold temperatures are prone to spoilage due to microbial action [35]. As these products are intended for direct consumption without any heat treatment prior to consumption, ensuring the safety of the product is imperative.

### 3.1. Development of a Chitosan Coating

**Figure 2:**
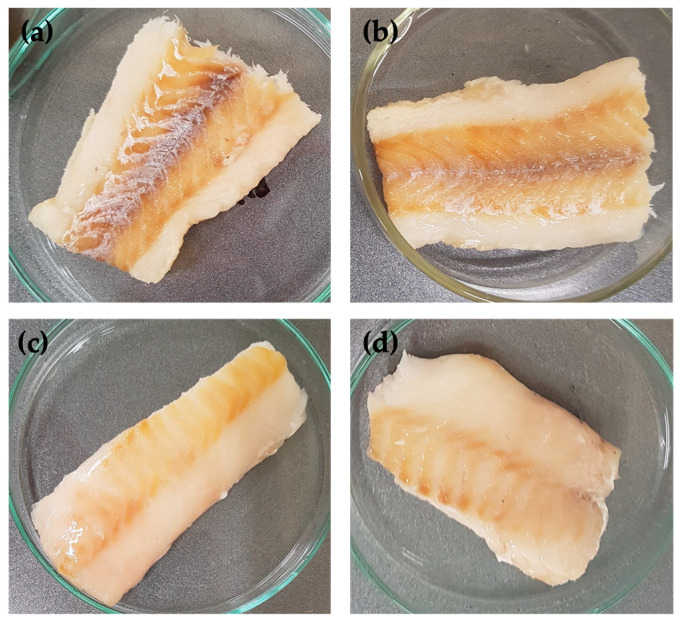
Uncoated fish sample (**a**), chitosan-coated fish sample (**b**), alginate crosslinked with calcium chloride-coated fish sample (**c**) and chitosan-alginate-crosslinked with calcium chloride-coated fish sample (**d**) stored for 3 days at 4 °C.

The visual assessment of the coating formulations and properties was conducted to evaluate their overall appearance. The chitosan FFS exhibited a clear, slightly yellowish appearance. Viscosity was found to be highly dependent on the chitosan concentration with higher concentrations resulting in increased viscosity. When applied to the RTE fish samples using the coating dipping method, all tested chitosan coatings adhered well to the sample surface producing a wet, shiny appearance (Figure 2). Following air drying during storage, no discernible differences between coated and uncoated samples were observed, indicating the invisibility of all tested coating formulations. Samples coated with 1% chitosan solution, showing a viscosity of 86 mPas, had a wet coating load of around 18.6 mg liquid coating/cm^2^. In comparison to alginate coating solutions, it was noted that higher viscosity increased the amount of coating adhering to the sample, which is a relationship supported in the literature [36]. The acidic solvent used for chitosan dissolution had a pH value of around 3.1, and the pH of the final chitosan FFSs was around 4.4. Previous studies showed that the low pH of the solvent alone did not adversely affect product quality [7].

**Figure 3:**
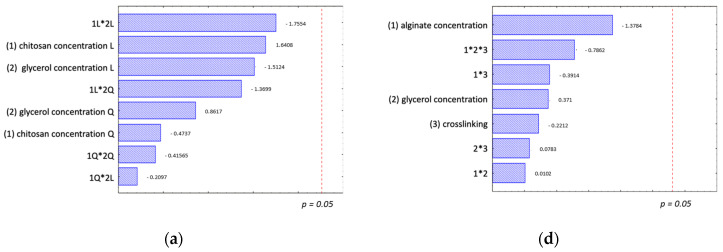

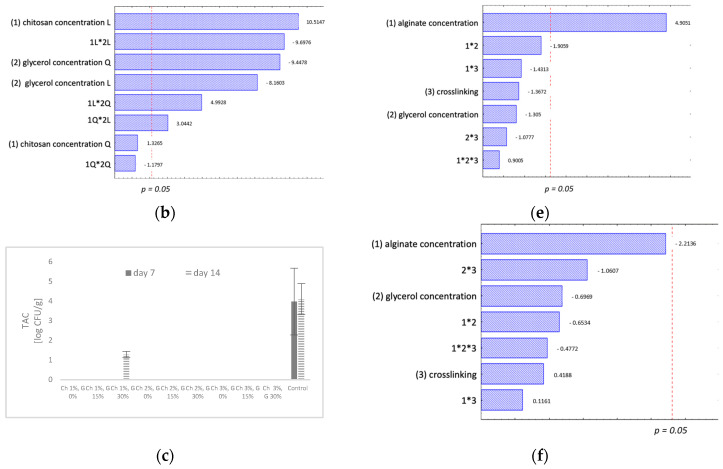
Statistical analysis of the screening experiment. Pareto charts of the effect of the chitosan concentration and glycerol concentration on (**a**) lipid oxidation and (**b**) water loss; and (**c**) total aerobic microbiological count after 14 days of storage at 4 °C. Pareto charts of the effect of alginate concentration, glycerol concentration and crosslinking with CaCl_2_ on (**d**) lipid oxidation, (**e**) water loss and (**f**) total aerobic count after 14 days of storage at 4 °C.

Overall, lipid oxidation increased across all samples during storage, yet even after 14 days of storage, it remained low with an average MDA value of 1.5 mg MDA eq./kg sample. According to the threshold criteria [37], these values classify the samples as ‘perfect quality material’ (values < 3 mg MDA eq./kg) suitable for human consumption. The statistical analysis showed that there was no significant differences between the tested coating formulations, as well as no significant differences between 7 and 14 days of storage in regard to lipid oxidation (Figure 3a). The identified variations in the selected design variables were within a similar range of magnitude as a recognised noise factor, which was potentially attributable to natural product variations. Consequently, it was not possible to distinguish between these factors. In conclusion, no discernible effect of the tested chitosan coating composition on controlling lipid oxidation in the RTE baked fish product could be observed within this experimental setup. These findings diverge from previous studies [10,38], which reported an observable effect of the tested chitosan coating in retarding oxidation when applied to a precooked beef patty or smoked sea bass fillet, respectively. One possible explanation for these different results could be that the samples in this study are less prone to lipid oxidation, or the effect may not have been apparent under the selected experimental conditions.

All samples experienced water loss during storage, which was driven by the gradient towards the surrounding air with a relative humidity of around 75%. Significant differences in the water loss between the tested coatings were observed. After 14 days of storage, the results from the full factorial design indicated that the variation in the dataset could be explained by the chitosan concentration, glycerol concentration, and the interactive effect between these two factors (Figure 3b). The lowest water loss occurred when applying a coating with a low chitosan concentration in combination with a medium glycerol concentration. As the chitosan concentration increased, water loss also increased, which is a trend mitigated by an increase in glycerol, up to 15% (*w*/*w* chitosan) in the coating. With higher glycerol concentrations (30% *w*/*w* chitosan), the combination with a higher chitosan concentration proved most effective in reducing water loss. Nevertheless, the lowest value obtained with 30% glycerol concentration was still higher than values with lower glycerol and chitosan concentrations. A similar effect of a chitosan coating, specifically a 2% (*w*/*v*) chitosan coating with 25% (*w*/*w* chitosan) glycerol, on the water loss of a precooked beef patty was reported [38].

All tested chitosan coatings showed a significant effect on the microbiological growth. The Total Aerobic Count (TAC) of the coated samples was below the detection limit after 14 days of storage at 4 °C except for samples coated with a 1% chitosan solution with 30% (*w*/*w* chitosan) glycerol addition, showing a TAC below 2 log CFU/g after 14 days (Figure 3c). Control samples showed a TAC of around 4 log CFU/g after 14 days stored at 4 °C. All tested chitosan coating formulations showed a similar effect on controlling the TAC of the RTE fish samples. Consequently, it can be inferred that the presence of glycerol in the solutions does not influence the antimicrobial effect of the chitosan, and a 1% chitosan concentration showed the same effect as the highest tested chitosan concentration of 3%. The reported antimicrobial effect of chitosan coatings in the literature aligns with the observed results. Previous studies have shown that a chitosan coating can retain good quality characteristics, improve microbiological safety, and extend shelf life during the storage of ready-to-cook meat products packed in LDPE pouches [7]. Additionally, the application of chitosan coatings with and without the incorporation of essential oils to RTE peeled shrimp tails under modified atmosphere packaging (MAP) conditions demonstrated the potential of chitosan coatings to maintain the microbiological quality [12]. Martínez et al. [10] studied the effect of a chitosan coating in combination with a vacuum package as a conservative method for smoked sea bass fillets and found that the coating inhibited microbiological growth. Sørbø und Lerfall [9] reported increased microbial stability and improve colour stability in RTE maki sushi when coated with chitosan and packed under low-CO_2_ MAP conditions.

In summary, based on the findings from all three quality parameters, a coating formulation with 1% chitosan and 15% (*w*/*w* chitosan) glycerol demonstrated the best performance in controlling the tested degradation processes during cold storage, which was primarily by reducing water loss and controlling microbiological growth.

### 3.2. Development of an Alginate Coating

The overall appearance of the coating formulations and coating properties were thoroughly investigated. The alginate FFSs exhibited a clear appearance and a consistent viscosity of around 9.43 mPas for all tested compositions. When applied to the RTE fish samples, using the coating dipping method, FFSs adhered to the sample surface. Crosslinking the alginate coating with calcium chloride resulted in a gel at the product surface, giving coated samples a wet, shiny appearance. Following air drying during storage, no discernible differences between coated and uncoated samples were observed, suggesting the invisibility of the coatings (Figure 2). The wet coating load of the alginate coatings was determined at around 10 mg liquid coating/cm^2^, indicating that a lower viscosity led to decreased coating adherence compared to the higher viscosity of the FFS of the chitosan coatings [7]. Statistical analysis results, evaluating the impact of alginate concentration, glycerol concentration, and crosslinking the alginate with CaCl_2_ on the lipid oxidation, water loss, and TAC of the baked fish sample are shown in Figure 3d–f.

An increase in malondialdehyde (MDA) value, indicative of fat oxidation, was observed for all tested alginate coated samples, reaching the highest value of around 2 mg MDA eq./kg for samples stored for 14 days. Under the assessed conditions, no tested factor on the alginate coating showed that the lipid oxidation was significant (Figure 3d), and no significant differences from control samples were found. A higher standard deviation within each coating formulation, may be attributed to natural variations in the tested baked fish samples. The tested factors of alginate concentrations, glycerol addition, or crosslinking with CaCl_2_ did not influence the performance of the alginate coating in providing an oxygen barrier to reduce lipid oxidation. Previous studies have reported a reduction in lipid oxidation for alginate-coated fish products during cold storage. Martínez et al. [10] found that an alginate coating, in combination with a vacuum package, inter alia, acted as a conservative method for smoked sea bass fillets, protecting against oxidation. Other studies with fresh fish fillet showed that with longer storage times, the use of an alginate coating reduced lipid oxidation [20,23]. However, in these studies, the lipid oxidation of the tested fish products was higher than in the present study. Therefore, the minimal detected effect could potentially be related to the low fat content of the RTE product, the overall low lipid oxidation of the product during the tested storage conditions, or it may not yet be visible under the selected storage conditions of 4 °C up to 14 days. It could be recommended to investigate the effect of the alginate coating on the lipid oxidation at higher storage temperatures to increase the lipid oxidation reaction rate or for longer storage times. In general, the product degradation concerning lipid oxidation could be more important for high fat content RTE food products.

Statistical results regarding the effect of the tested alginate coatings on the moisture loss are presented in Figure 3e. Moisture loss increased with longer storage times, and the moisture loss rate depended on the coating formulation. The full factorial design results indicated that the alginate concentration factor was significant after 14 days of storage, highlighting the importance of a lower alginate concentration to decrease the water loss. For shorter storage times, the interactive effect between alginate concentration and glycerol concentration was also significant. However, for storage times up to 14 days, this interactive effect was only marginally significant. When the alginate concentration increased, the glycerol concentration needed to increase for better control of the water loss during cold storage. The third tested factor crosslinking of the alginate coating with CaCl_2_ was not significant. Considering the impact of the coatings on the water loss, a 1% alginate concentration without glycerol and no crosslinking emerged as the preferred choice for a RTE seafood product.

Statistical results regarding TAC are shown in Figure 3f. Microbiological growth increased during storage, reaching an average TAC of around 4 log CFU/g sample after 7 days and around 7 log CFU/g sample after 14 days. These results are within the same range as the control sample without any coating. Statistical analysis of the results showed no significant differences between the tested alginate coating formulations after 7 and 14 days stored at 4 °C. After 14 days of storage, only a marginal effect of the alginate concentration was observed, indicating that an alginate concentration of 2% was marginally significant for reducing the TAC. Different findings of the effect of alginate coating on microbiological growth were presented in the literature. Some studies reported a reduction in microbiological growth for samples coated with sodium alginate due to the ability of the coating to act as a barrier against oxygen transfer [22], and this effect could be increased by the use of additives [20,21]. Nevertheless, other researchers reported no reduction in microbial growth [10,23,39].

In summary, considering the findings for all the tested quality parameters, a coating formulation with 1% alginate without glycerol and no crosslinking was recommended to enhance the product quality. While the alginate coating demonstrated good film-forming properties, its effect on lipid oxidation, water loss and TAC in RTE baked fish products is limited and could possibly be enhanced by the inclusion of natural additives as antioxidants or antimicrobials.

### 3.3. Comparing the Performance of a Chitosan, Alginate and Bilayer Coating under Optimal and Abused Storage Conditions

A follow-up experiment was conducted to further investigate and compare the properties of the selected chitosan and alginate coatings. The combination of a 1% chitosan solution with 15% glycerol (*w*/*w* chitosan) and 1% alginate coating was previously identified to be the most suitable for the RTE fish product under study. In addition, the potential of synergistic effects of combining the individual coating properties of the chitosan and alginate coating were explored by applying a bilayer coating to the RTE baked fish fillet. A full factorial experimental design was employed to study the effect of these coatings under optimal storage conditions at 4 °C and abusive conditions of 14 °C, over 21 days and 7 days, respectively (Table 2). The effect of the storage temperature was investigated after 7 days of storage.

All tested samples, including the chitosan-coated, alginate-coated, and bilayer-coated samples, and an untreated sample as control, showed a significant increase in MDA value over the tested storage time, indicating lipid oxidation. However, the highest value measured over the tested storage conditions, reaching 4.4 mg MDA eq./kg for uncoated and alginate-coated samples stored at 4 °C for 21 days, still fell within the range considered acceptable for good quality material based on suggested threshold criteria [37]. The results showed that even for longer storage times of up to 21 days for samples stored at 4 °C and up to 7 days for samples stored at 14 °C, lipid oxidation did not emerge as a critical quality parameter for the studied product. The statistical analysis revealed no significant differences between the control, chitosan, alginate or a double coating at each sampling day regarding lipid oxidation (Table 2). 

**Figure 4:**
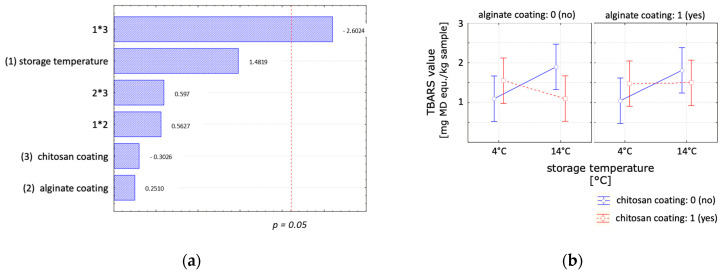
Statistical analysis of the effect of the chitosan coating, alginate coating and storage temperature on the lipid oxidation. (**a**) Pareto chart and (**b**) marginal means plot with a confident limit of 95% at day 7.

When investigating the effect of the storage temperature, a significant interactive effect between storage temperature and chitosan coating was observed (Figure 4). As anticipated, a slightly higher increase in lipid oxidation for samples stored at 14 °C compared to those stored at 4 °C was observed. At higher storage temperatures, samples coated with chitosan showed a lower lipid oxidation. These findings align with other studies that reported no significant effect for short storage times and a slight effect for longer storage times of chitosan coatings on the lipid oxidation of RTE meat products. Wu et al. [38] reported that a chitosan coating on precooked beef patties stored for 3 days at 4 °C showed no significant effect in reducing TBARS compared to an uncoated, unpacked sample. Kanatt et al. [7] reported that chitosan-coated ready-to-cook meat products displayed a longer shelf life compared to uncoated samples, as the tested chitosan coating slightly retarded lipid oxidation in all the meat products during storage. 

No significant effect of the alginate coating regarding storage temperature was identified. However, a bilayer coating demonstrated reduced lipid oxidation at higher storage temperatures, which was most likely due to the positive effect of the chitosan layer of the bilayer coating.

**Figure 5:**
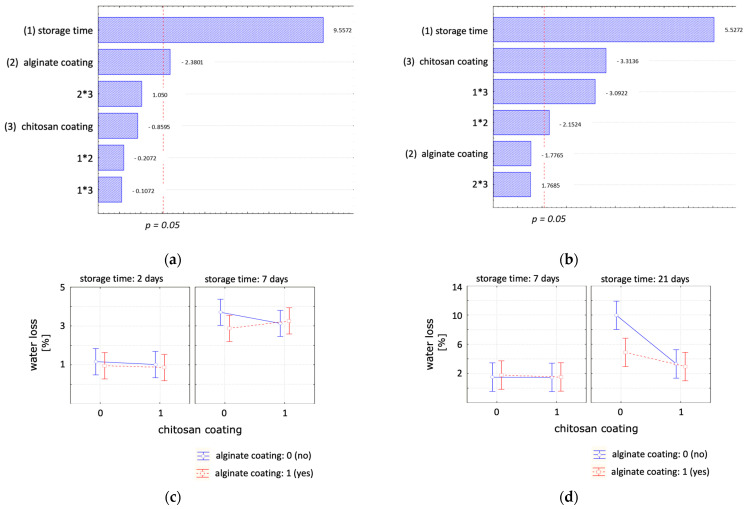
Statistical analysis of the effect of chitosan coating, alginate coating and storage time on the moisture loss at (**a**,**c**) 14 °C and (**b**,**d**) 4 °C. (**a**,**c**) Pareto charts, (**b**,**d**) plot of marginal means with a confident limit of 95%.

The impact of chitosan, alginate, and a bilayer coating, in comparison to an uncoated control sample, on the product water loss under optimal storage temperature of 4 °C and abusive storage temperature of 14 °C was investigated, and the results are presented in Table 2. Overall, a reduction in water loss was observed for all the scenarios studied. The influence of the coating treatments on weight loss was examined at 14 °C (Figure 5a,c) and 4 °C (Figure 5b,d). For samples stored at 14 °C, only the alginate coating exhibited significance with a slight reduction in water loss over time. The chitosan coating, the interactive effect between factors ‘storage time’ and ‘chitosan coating’ and the interactive effect between the factors ‘alginate coating’ and ‘storage time’ were significant for samples stored under cooled conditions. The applied coatings reduced water loss for extended storage durations. When studying the storage temperature effect after 7 days, storage temperature was the only significant parameter, exerting a high effect on weight loss. The average moisture loss after 7 days stored at 14 °C was 3.2%, while at 4 °C, it was 1.6%. 

In conclusion, the tested coating formulations effectively mitigated weight loss in the samples during storage. Samples, whether coated with a single chitosan coating or a double coating, exhibited weight losses of around 3.3 and 3% after 21 days of storage compared to the control sample with a 10% weight loss. However, with the double coating, it was not possible to synergise the effects of the chitosan and the single alginate coating.

In this study, the TAC was used to evaluate the overall safety and quality of the RTE fish product. The observed results of the TAC of the RTE fish samples are presented in Table 2. The initial TAC of freshly baked uncoated RTE fish product fell within the range of 1 log CFU/g sample. Generally, the TAC increased with storage time, although the growth rate could be significantly influenced by the storage temperature and the application of a coating to the product. 

**Figure 6:**
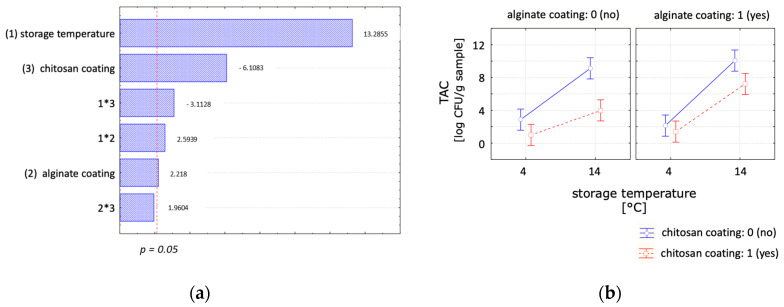
Statistical analysis of the effect of the chitosan coating, alginate coating and storage temperature on total aerobic count. (**a**) Pareto chart and (**b**) marginal means plot with a confident limit of 95% at day 7.

The microbiological growth rate decreased with lower temperature, as evidenced by reaching the maximum allowed microbiological load of 7 log CFU/g per sample at 14 °C after 4 days and at 4 °C after 14 days for uncoated control samples. Following storage temperature, the chitosan coating emerged as the second significant parameter (Figure 6). The results indicated that the microbial growth during storage could be reduced with the application of a chitosan coating. The chitosan-coated samples remained below the critical value of 7 log CFU/g during the tested storage times of 7 days at 14 °C and 21 days at 4 °C. Conversely, the alginate coating showed a negative effect on the microbial growth. With longer storage times, samples coated with alginate resulted in higher CFU values compared to the control samples. Meanwhile, samples coated with both chitosan and alginate showed a reduced microbial growth when compared to the uncoated samples; the former showed a higher CFU count than those with a single chitosan coating. These findings align with other studies on chitosan coatings, which reported an antimicrobial effect of chitosan [8,10,11,13]. The impact of chitosan on microbial stability in RTE maki sushi during storage under abused conditions has been previously reported [9], emphasising an enhanced safety profile for RTE products not only during an optimal refrigerated chain but also when exposed to adverse conditions. Therefore, it is recommended to conduct further investigations to assess the efficacy of a chitosan coating under simulated real shelf-life conditions.

### 3.4. Effect of the Edible Coatings on the Product Shelf Life

A multi-response analysis was employed to assess the shelf life of both uncoated and coated products with the shelf life estimation criteria set at a TAC (Total Aerobic Count) of 7 log CFU/g (according to the Food Safety Authority of Ireland 2020), a maximum 10% water loss, and lipid oxidation value of below 8 mg MD eq./kg sample [37]. The results, reflecting the total desirability score of the RTE fish products, where each quality parameter was weighted equally without comprising for another, are summarised in Table 3. Overall, the chitosan coating consistently yielded the highest score at each storage time and temperature, maintaining an acceptable quality score for up to 21 and 7 days when stored at 4 and 14 °C, respectively. The second-best performer was a bilayer coating, albeit with a diminished quality and consequently a shorter maximal shelf life. The alginate coating offered only marginal advantages in terms of prolonging quality. A noteworthy drawback of the alginate coating, evident across all three tested quality factors, was its adverse impact on the aerobic microbial growth.

## 4. Conclusions

Edible coatings represent an eco-friendly alternative in packaging systems, utilising biobased materials to control the degradation processes of food products. They offer enhanced food safety and quality. In this study, coatings formulated with chitosan and alginate were examined for their effectiveness in managing fat oxidation, water loss, and microbial growth in an RTE baked fish product. Initially, the study examined the impact of changes in the coating composition, which was followed by an analysis of the developed coatings’ effect on the product shelf life under both optimal and abused storage conditions. The results demonstrated that the selected coating composition significantly influences coating performance. A 1% (*w*/*v*) chitosan concentration proved sufficient to control the microbial growth, and it was preferable for managing water loss in the samples. An interactive effect was observed between the chitosan concentration and glycerol concentration in the coating solutions. The most effective coating solution for the RTE fish product of this study comprised 1% (*w*/*v*) chitosan, 15% glycerol (*w*/*w* chitosan), and a 1% (*w*/*v*) sodium alginate coating. Both chitosan and alginate coatings whether applied individually or as a bilayer formed discreet, adherent layers around the product. The tested alginate coatings showed a non-significant impact on the quality parameters of the RTE seafood product. In contrast, the chitosan coating demonstrated a significant reduction in microbial growth and a decrease in weight loss during storage at both optimal and abused temperatures. The application of a chitosan coating extended shelf life from 7 to 21 days under optimal storage conditions at 4 °C and from 2 to 7 days under abused conditions. The observed effects of the tested coatings, under both optimal and abused storage conditions, highlight the viability of using edible coatings as a safety measure to mitigate temperature fluctuation throughout the food supply chain. These findings underscore the efficacy of a chitosan coating as an effective packaging strategy to enhance the safety and quality of an RTE seafood product during cold storage and under fluctuating storage conditions.

## Figures and Tables

**Table 1:** Overview of experimental designs for (a) development of a chitosan coating, (b) development of an alginate coating and (c) comparing the coating performances of alginate and chitosan coating at optimal and abused storage temperature.

Experimental Set-Up	Factors	Levels					
(a)	Chitosan concentration % (*w*/*v*)	1		2		3	
	Glycerol concentration % (*w*/*w* chitosan)	0		15		30	
(b)	Alginate concentration % (*w*/*v*)	1			2		
	Glycerol concentration % (*w*/*w* alginate)	0			15		
	Crosslinking with calcium chloride	yes			no		
(c)	Storage temperature	4			14		
	Alginate coating	no			yes		
	Chitosan coating	no			yes		

**Table 2:** Measured quality parameters (lipid oxidation, water loss and total aerobic count) of samples coated with a single alginate, a single chitosan and a bilayer chitosan–alginate coating stored at (a) 14 °C over 7 days and (b) 4 °C over 21 days.

**Lipid Oxidation (mg MDA eq./kg Sample)**				
(a)	day 0	day 2	day 4	day 7
Control	0.16 ± 0.03 a,A	0.54 ± 0.53 a,A	0.66 ± 0.27 a,A,B	1.90 ± 0.82 a,B
Alginate	0.16 ± 0.03 a,A	0.79 ± 0.22 a,A	1.56 ± 0.91 a,A	1.10 ± 0.10 a,B
Chitosan	0.16 ± 0.03 a,A	0.28 ± 0.17 a,A,B	0.50 ± 0.12 a,B	1.81 ± 0.38 a,A,B
Chitosan + Alginate	0.16 ± 0.03 a,A	0.54 ± 0.07 a,B	1.15 ± 0.11 a,C	1.50 ± 0.03 a,D
(b)	day 0	day 7	day 14	day 21
Control	0.16 ± 0.03 a,A	1.10 ± 0.62 a,A	1.17 ± 0.39 a,A	4.38 ± 1.20 a,B
Alginate	0.16 ± 0.03 a,A	1.55 ± 0.17 a,A,B	2.21 ± 0.50 b,A,B,C	4.36 ± 0.96 a,C
Chitosan	0.16 ± 0.03 a,A	1.05 ± 0.38 a,B	2.40 ± 0.42 a,b,B	4.22 ± 1.09 a,C
Chitosan + Alginate	0.16 ± 0.03 a,A	1.48 ± 0.66 a,A,B	1.75 ± 0.42 a,b,A,B	2.07 ± 0.89 a,A,B
**Water Loss (%)**				
(a)		day 2	day 4	day 7
Control		1.16 ± 0.35 a,A	2.70 ± 0.44 a,B	3.70 ± 0.32 a,B
Alginate		0.96 ± 0.17 a,A	1.78 ± 0.36 a,A,B	2.87 ± 0.54 a,B
Chitosan		1.02 ± 0.31 a,A	2.32 ± 0.66 a,A	3.13 ± 1.00 a,A
Chitosan + Alginate		0.86 ± 0.06 a,A	1.59 ± 0.11 a,A	3.26 ± 0.52 a,B
(b)		day 7	day 14	day 21
Control		1.50 ± 0.49 a,A	3.65 ± 0.98 a,A	9.96 ± 4.02 a,A
Alginate		1.77 ± 0.25 a,A	2.63 ± 0.30 a,A,B	4.88 ± 0.76 a,b,B
Chitosan		1.46 ± 0.30 a,A	1.99 ± 0.28 a,A	3.31 ± 0.50 a,b,B
Chitosan + Alginate		1.52 ± 0.68 a,A	2.28 ± 0.51 a,A	2.94 ± 0.71 b,A
**Total Aerobic Count (log CFU/g Sample)**				
(a)	day 0	day 2	day 4	day 7
Control	<1.06 * ± 0.10 a,A	4.86 ± 0.74 a,B	7.19 ± 0.58 a,b,C	9.11 ± 0.45 a,D
Alginate	<1.06 * ± 0.10 a,A	5.85 ± 0.05 a,B	7.70 ± 0.58 b,C	10.06 ± 0.06 a,D
Chitosan	<1.06 * ± 0.10 a,A	<1.39 * ± 0.67 b,A	<0.94 * ± 0.34 c,A	5.07 ± 2.56 b,A
Chitosan + Alginate	<1.06 * ± 0.10 a,A	2.79 * ± 0.74 b,A	6.00 ± 0.90 a,B	7.21 * ± 1.05 a,b,B
(b)	day 0	day 7	day 14	day 21
Control	<1.06 * ± 0.10 a,A	<2.86 * ± 0.84 a,A	7.63 ± 0.75 a,B	9.10 ± 1.04 a,B
Alginate	<1.06 * ± 0.10 a,A	<2.13 * ± 0.23 a,b,B	>8.48 * ± 0.00 a,C	10.35 ± 0.41 a,D
Chitosan	<1.06 * ± 0.10 a,A	<1.00 * ± 0.00 b,A	<1.00 * ± 0.00 b,A	<1.79 * ± 2.29 b,A
Chitosan + Alginate	<1.06 * ± 0.10 a,A	<1.40 * ± 0.70 a,b,A	4.85 * ± 2.81 a,A	10.06 ± 0.00 a,B

* Statistical analyses were conducted for samples at 14 °C (a) and 4 °C (b) separately. For each storage temperature, results on the same column followed by the same small letter and results in the same row followed by the same capital letter were not statistically different (*p* < 0.05) according to Tukey’s HSD test. * indicates an estimated value due at least one colony count of the triplicates below 30 or above 300. >/< indicates the direction to the potential real TAC value.

**Table 3:** Overall desirability score for the quality of tested RTE baked fish samples coated and uncoated.

		Day 0	Day 2	Day 4	Day 7	Day 14	Day 21	Day 28
4 °C	Alginate	1			0.84	0	0	0
	Chitosan	1			0.89	0.84	0.65	0
	Chitosan-Alginate	1			0.87	0.61	0	0
	Control	1			0.80	0	0	0
14 °C	Alginate	1	0.56	0	0	0	0	0
	Chitosan	1	0.92	0.86	0.67	0	0	0
	Chitosan-Alginate	1	0.85	0.50	0	0	0	0
	Control	1	0.67	0	0	0	0	0
			1	0.8	0.6	0.4	0.2	0
			colour scale from 1 to 0					

## Data Availability

Data are contained within the article.
